# Electrophysiological Profile of Different Antiviral Therapies in a Rabbit Whole-Heart Model

**DOI:** 10.1007/s12012-024-09872-3

**Published:** 2024-06-08

**Authors:** Julian Wolfes, Lina Kirchner, Florian Doldi, Felix Wegner, Benjamin Rath, Lars Eckardt, Christian Ellermann, Gerrit Frommeyer

**Affiliations:** Department of Cardiology II (Electrophysiology), University Hospital Münster, Albert-Schweitzer-Campus 1, 48149 Münster, Germany

**Keywords:** Hydroxychloroquine, Azithromycin, Remdesivir, Lopinavir, Long QT syndrome, Arrhythmia, Sudden cardiac death, Anitviral

## Abstract

Antiviral therapies for treatment of COVID-19 may be associated with significant proarrhythmic potential. In the present study, the potential cardiotoxic side effects of these therapies were evaluated using a Langendorff model of the isolated rabbit heart. 51 hearts of female rabbits were retrogradely perfused, employing a Langendorff-setup. Eight catheters were placed endo- and epicardially to perform an electrophysiology study, thus obtaining cycle length-dependent action potential duration at 90% of repolarization (APD_90_), QT intervals and dispersion of repolarization. After generating baseline data, the hearts were assigned to four groups: In group 1 (HXC), hearts were treated with 1 µM hydroxychloroquine. Thereafter, 3 µM hydroxychloroquine were infused additionally. Group 2 (HXC + AZI) was perfused with 3 µM hydroxychloroquine followed by 150 µM azithromycin. In group 3 (LOP) the hearts were perfused with 3 µM lopinavir followed by 5 µM and 10 µM lopinavir. Group 4 (REM) was perfused with 1 µM remdesivir followed by 5 µM and 10 µM remdesivir. Hydroxychloroquine- and azithromycin-based therapies have a significant proarrhythmic potential mediated by action potential prolongation and an increase in dispersion. Lopinavir and remdesivir showed overall significantly less pronounced changes in electrophysiology. In accordance with the reported bradycardic events under remdesivir, it significantly reduced the rate of the ventricular escape rhythm.

## Introduction

In the wake of the COVID-19 pandemic, numerous antiviral therapies have been and are still being tested. Most of the preparations used are off-label and have already been approved for other indications. Antimalarial and antiretroviral agents are drugs used in this context [1].

Especially at the beginning of the pandemic, the use of hydroxychloroquine and azithromycin was discussed to mitigate severe courses of COVID-19 ARDS [2]. Subsequently, lopinavir [3] and remdesivir [4] were used. While the first three did not show any superiority to placebo and were in part associated with poorer clinical outcomes [1, 5, 6], remdesivir was also convincing in a large randomized controlled trial and continues to be used to treat COVID-19 [4] while the others are still used as antimalarials, antibiotics and antiretroviral therapies. Proarrhythmic effects were repeatedly described with the use of the different preparations. Although there are also diverging reports on the possible proarrhythmic potential of the various preparations.

Hydroxychloroquine shows a potential proarrhythmic potential through the inhibition of hERG channels [7], although the clinical proarrhythmic potential of hydroxychloroquine appears to be rather low [8, 9]. The combination of azithromycin and hydroxychloroquine showed a significant arrhythmic risk in some studies [10, 11], although the proarrhythmic potential of sole azithromycin appears to be rather low, both in clinical practice [12] and in previous studies by our group [13].

Although clinical data on possible proarrhythmic effects of lopinavir are scarce, preclinical data show a suspected hERG [14] inhibition with possible proarrhythmic capability.

There are also contradictory data on remdesivir regarding the risk of arrhythmia. For example, there are diverging data on any hERG inhibition [15, 16]. Of particular clinical relevance is the tendency to bradycardia observed with remdesivir [17–19], although the underlying mechanisms have not been conclusively clarified.

Since all the above-mentioned drugs are still widely used in the clinic, although partly not for the treatment of COVID-19, the aim of the present study was to characterize their electrophysiological safety profile in an established model of the isolated rabbit heart.

## Methods

The experimental protocol was approved by the local animal care committee and the local federal institution (Landesamt für Natur, Umwelt und Verbraucherschutz Nordrhein-Westfalen, File number: 81–02.05.50.21.004).

The experimental Langendorff whole-heart setup has been extensively described by our group in previous publications [20–22].

In summary, 51 rabbit hearts were harvested from female New Zealand White rabbits, aged 12–24 weeks, after the animals were euthanized by exsanguination. The hearts were retrogradely perfused in a Langendorff apparatus with warmed and oxygenated (95% O_2_, 5% CO_2_) Krebs–Henseleit buffer (NaCl 118 mM, NaHCO_3_ 24.88 mM, D-glucose 5.55 mM, KCl 4.70 mM, Na-pyruvate 2 mM, CaCl_2_ 1.80 mM, KH_2_PO_4_ 1.18 mM, MgSO_4_ 0.83 mM).

To detect monophasic action potentials seven catheters were placed epicardially and one endocardial catheter was placed in the left ventricle. A 12 lead ECG was recorded form the warming-bath surrounding the heart.

Mechanical AV nodal ablation was performed in the atrioseptal region. Thereafter, the following protocol was employed:

First, the unstimulated ventricular escape rate of the hearts was determined. Subsequently, pacing was performed with seven different cycle lengths between 900 and 300 ms while the cycle length-dependent monophasic action potentials and QT intervals were determined.

**Fig. 1:**
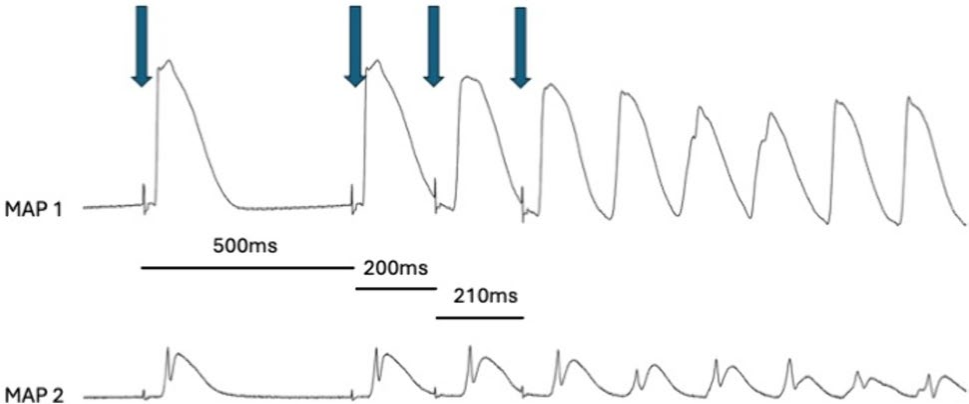
Representative example of ventricular tachycardia arising from programmed ventricular stimulation (arrows).

Subsequently, programmed ventricular pacing with a short-coupled extrastimulus was performed to determine the effective refractory period (ERP). In addition, repetitive burst stimulations were used to record ventricular vulnerability. An example of ventricular tachycardia induces by burst stimulation is given in Fig. 1. This was followed by perfusion with hypokalemic KHB (K + 1.5 mM) to determine arrhythmia susceptibility in a hypokalemic environment.

The action potential duration at 90% of repolarization (APD_90_) was measured between the fastest upstroke and 90% of repolarization of the action potential. Spatial dispersion of repolarization was determined by the difference of the maximum and the minimum of the eight simultaneously recorded monophasic action potentials. Post repolarization refractoriness (PRR) was calculated as the difference between ERP and APD_90_.

The 51 hearts were allocated to four groups with 12 to 13 animals each group (*n* = 13): In group 1 (HXC), 13 hearts were treated with 1 µM hydroxychloroquine. Thereafter, 3 µM hydroxychloroquine was infused. Group 2 (HXC + AZI) 13 hearts were perfused with 3 µM hydroxychloroquine followed by 150 µM azithromycin. In group 3 (LOP), the 12 hearts were perfused with 3 µM lopinavir followed by 5 µM and 10 µM lopinavir. Group 4 (REM) 13 hearts were perfused with 1 µM remdesivir followed by 5 µM and 10 µM remdesivir. The sample size was calculated according to previous studies [20].

Statistics.

Values are shown as mean ± standard deviation. Statistical analyses and graphic visualizations were performed employing Graphpad Prism Version 9. Drug effects on APD_90_, QT interval, spatial dispersion of repolarization and effective refractory periods were analysed employing repeated measures ANOVA for multiple comparisons. P values < 0.05 were considered to be statistically significant.

## Results

### Hydroxychloroquine

Hydroxychloroquine did not significantly alter the APD_90_ (baseline: 153 ± 32 ms; 1 µM HXC: 154 ± 25 ms, *p* = ns; 3 µM HXC: 160 ± 28 ms *p* = ns) while the higher dose of 3 µM HXC induced a small but significant prolongation of the QT interval (baseline: 274 ± 38 ms; 1 µM HXC: 272 ± 49 ms, *p* = ns; 3 µM HXC: 282 ± 41 ms p < 0.05). Spatial dispersion of repolarization was not significantly influenced in the presence of hydroxychloroquine (baseline: 50 ± 22 ms; 1 µM HXC: 46 ± 19 ms, *p* = ns; 3 µM HXC: 56 ± 36 ms *p* = ns). The effective refractory period was not significantly altered under hydroxychloroquine while 1 µM shortened the PRR and 3 µM hydroxychloroquine caused a restitution of the baseline PRR.

**Fig. 2:**
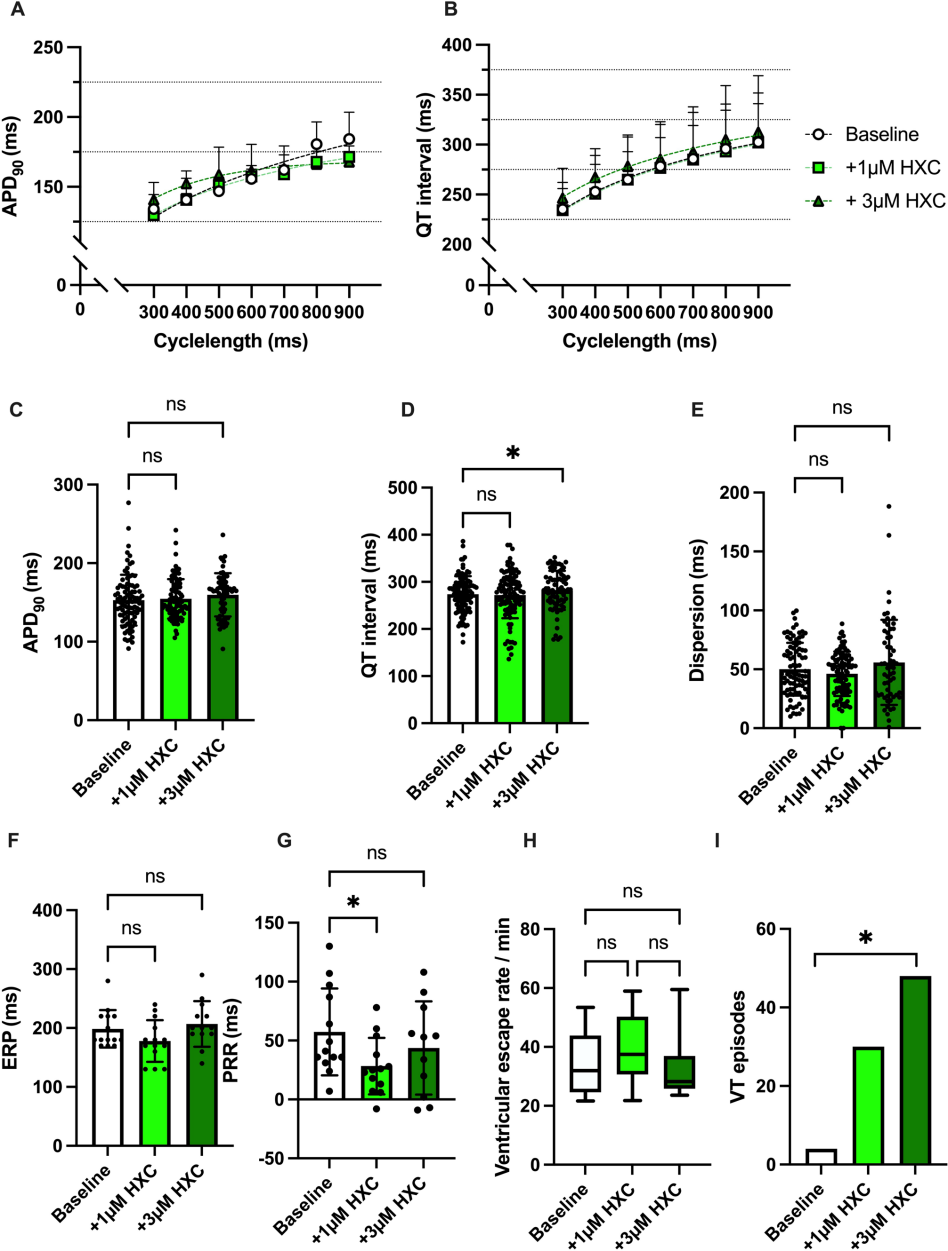
**A** Cycle length-dependent action potential durations (APD_90_) and **B** QT intervals under baseline conditions (empty circles) and after treatment with 1 µM (green square), 3 µM (dark green triangle) hydroxychloroquine (HXC). **C** Overall APD_90_ and **D** QT interval. Concentration-dependent effect of hydroxychloroquine on **E** spatial dispersion of repolarization, **F** effective refractory period (ERP), **G** post-repolarization refractoriness (PRR) and **H** ventricular escape rate. **I** Number of ventricular tachycardia (VT)/ fibrillation (VF) induced by programmed ventricular stimulation (* = p<0.05).

The incidence of ventricular arrhythmia episodes increased under perfusion with hydroxychloroquine reaching statistical significance with the highest concentrations of 3 µM (baseline: 4 episodes; 1 µM HXC: 30 episodes, *p* = ns; 3 µM HXC: 48 episodes, p < 0.05). The ventricular escape rhythm was not significantly influenced by the perfusion with hydroxychloroquine. The effects of hydroxychloroquine on cardiac electrophysiology are summarized in Fig. 2.

### Combination of Hydroxychloroquine and Azithromycin

**Fig. 3:**
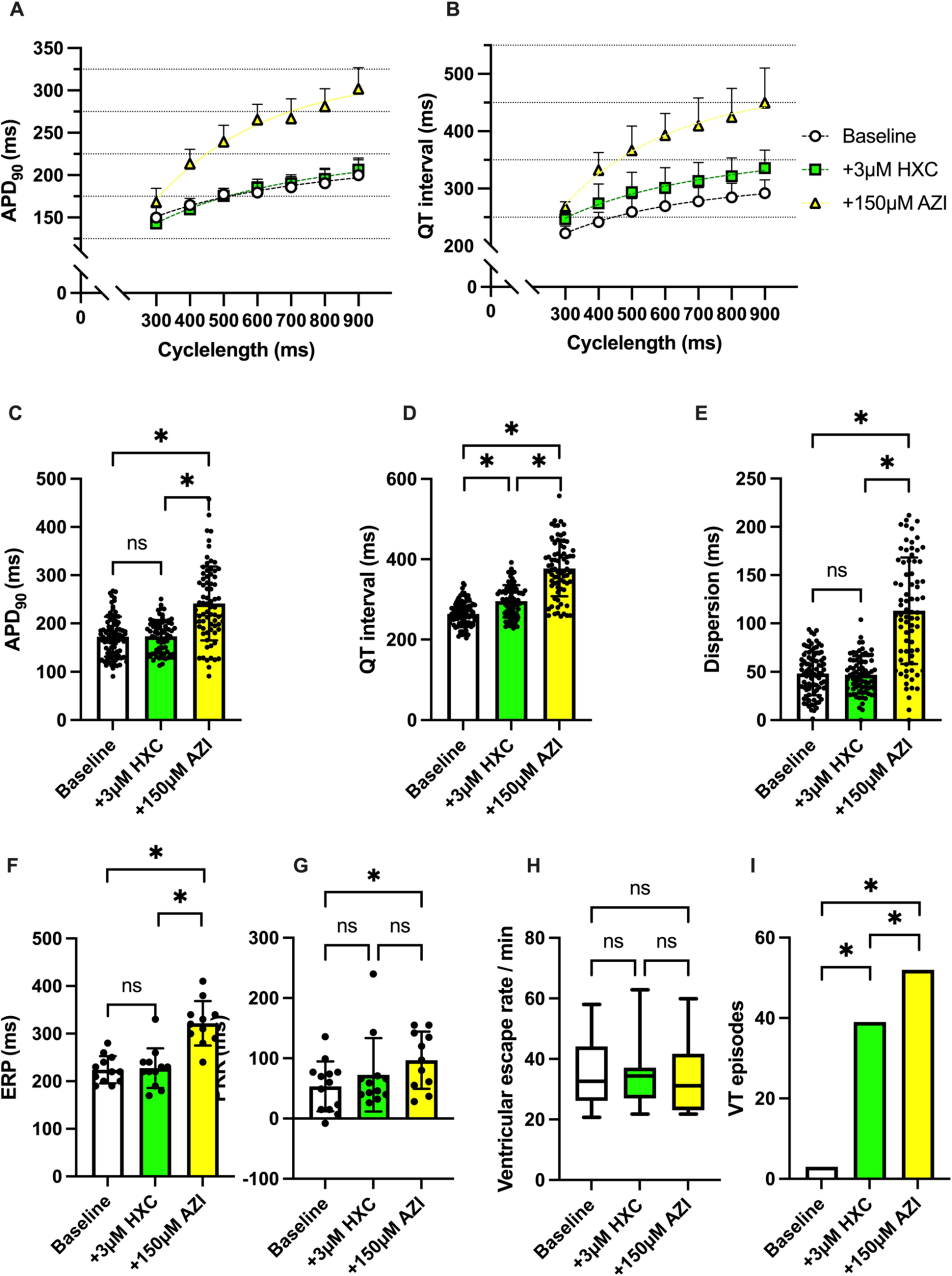
**A** Cycle length-dependent action potential durations (APD_90_) and **B** QT intervals under baseline conditions (empty circles) and after treatment with 3 µM hydroxychloroquine (HXC) (green square) and additional perfusion with 150 µM (yellow triangle) azithromycin (AZI). **C** Overall APD_90_ and **D** QT interval. Effect of hydroxychloroquine and azithromycin on **E** spatial dispersion of repolarization, **F** effective refractory period (ERP), **G** post-repolarization refractoriness (PRR) and **H** ventricular escape rate. **I** Number of ventricular tachycardia (VT)/ fibrillation (VF) induced by programmed ventricular stimulation (* = p<0.05).

The combination of hydroxychloroquine and azithromycin caused a significant prolongation of QT interval (baseline: 264 ± 31 ms; 3 µM HXC: 295 ± 40 ms, p < 0.05; 3 µM HXC + 150 µM AZI: 377 ± 69 ms p < 0.05) and APD_90_ (baseline: 172 ± 42 ms; 3 µM HXC: 173 ± 34 ms, *p* = ns; 3 µM HXC + 150 µM AZI: 241 ± 77 ms p < 0.05). Furthermore, the spatial dispersion of repolarization was significantly altered when combining hydroxychloroquine and azithromycin (baseline: 48 ± 22 ms; 3 µM HXC: 47 ± 19 ms, *p* = ns; 3 µM HXC + 150 µM AZI: 113 ± 55 ms p < 0.05). Effective refractory period and post-repolarization refractoriness were prolonged under additional perfusion with azithromycin. Arrhythmia incidence was significantly increased when adding azithromycin to hydroxychloroquine (baseline: 3 episodes; 3 µM HXC: 39 episodes, p < 0.05; 3 µM HXC + 150 µM AZI: 52 episodes, p < 0.05). Ventricular escape rhythm was not significantly influenced by the perfusion with hydroxychloroquine and azithromycin. The effects of hydroxychloroquine and azithromycin on cardiac electrophysiology are summarized in Fig. 3.

### Lopinavir

**Fig. 4:**
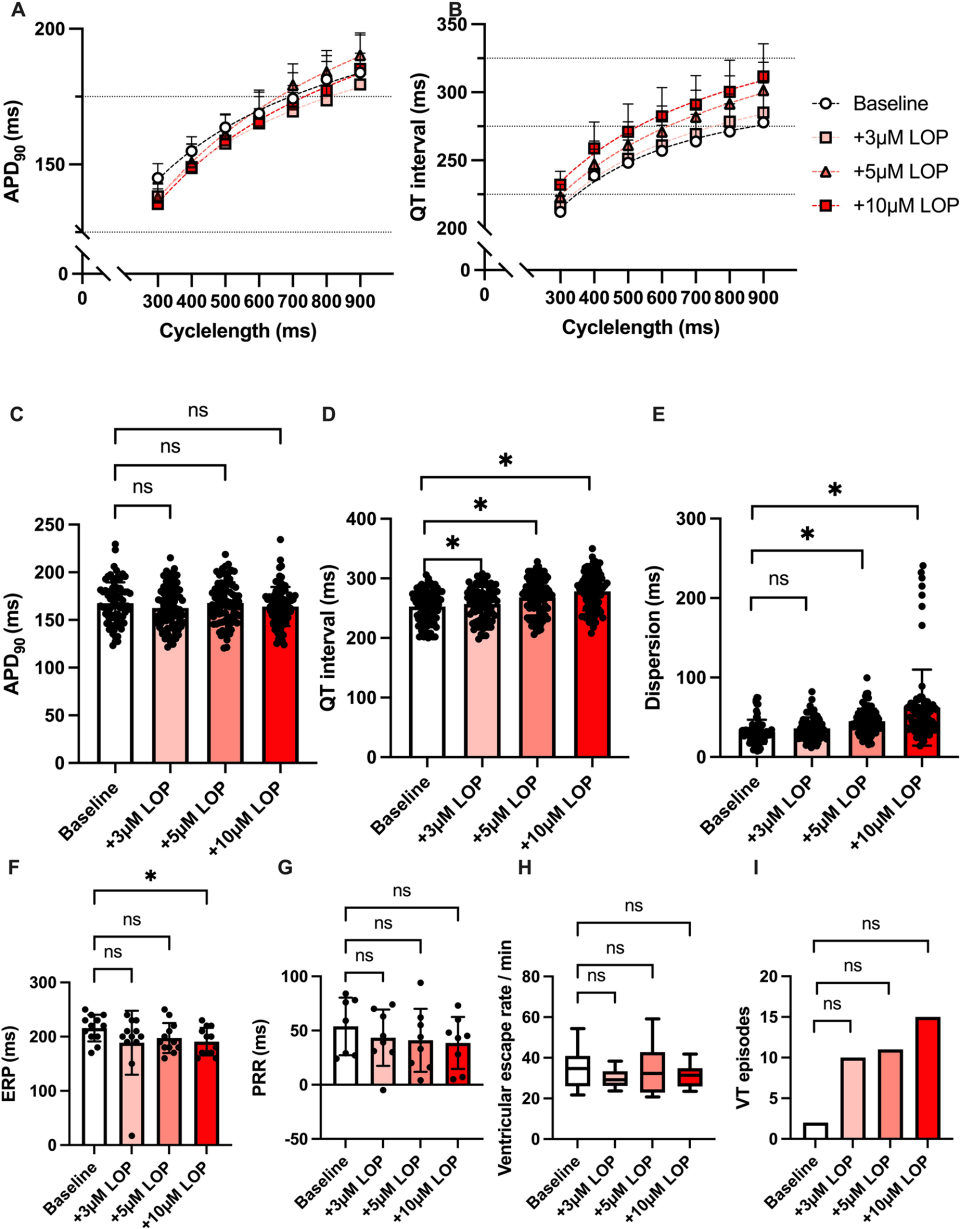
**A** Cycle length-dependent action potential durations (APD_90_) and **B** QT intervals under baseline conditions (empty circles) and after treatment with 3 µM (pink square), 5 µM (red triangle) and 10µM (red square) lopinavir (LOP). **C** Overall APD_90_ and **D** QT interval. Concentration-dependent effect of lopinavir on **E** spatial dispersion of repolarization, **F** effective refractory period (ERP), **G** post-repolarization refractoriness (PRR) and **H** ventricular escape rate. **I** Number of ventricular tachycardia (VT)/ fibrillation (VF) induced by programmed ventricular stimulation (* = p<0.05).

Perfusion with lopinavir did not significantly alter APD_90_ (baseline: 168 ± 22 ms; 3 µM LOP: 163 ± 22 ms, *p* = ns; 5 µM LOP: 168 ± 22 ms, *p* = ns; 10 µM LOP: 164 ± 20 ms, *p* = ns) while a small but significant prolongation of the QT interval (baseline: 253 ± 27 ms; 3 µM LOP: 257 ± 29 ms, p < 0.05; 5 µM LOP: 268 ± 30 ms, p < 0.05; 10 µM LOP: 278 ± 32 ms, p < 0.05) could be observed. The dispersion of repolarization was increased when employing high doses of lopinavir (baseline: 32 ± 15 ms; 3 µM: LOP: 36 ± 14 ms, *p* = ns; 5 µM LOP: 45 ± 16 ms, p < 0.05; 10 µM LOP: 62 ± 48 ms, p < 0.05). ERP and PRR were overall not significantly altered under lopinavir while only high doses of lopinavir induced a slight shortening of the ERP. Furthermore, the incidence of VT episodes was slightly and non-significantly raised under perfusion with lopinavir (baseline: 2 episodes; 3 µM: LOP: 10 episodes, *p* = ns; 5 µM LOP: 11 episodes, *p* = ns; 10 µM LOP: 15 episodes, *p* = ns). The ventricular escape rhythm was not significantly influenced by perfusion with lopinavir. The effects of lopinavir on cardiac electrophysiology are summarized in Fig. 4.

### Remdesivir

Perfusion with remdesivir caused a contrary effect on APD_90_ and QT interval with significant QT interval prolongation (baseline: 254 ± 32 ms; 1 µM REM: 263 ± 35 ms, p < 0.05; 5 µM REM: 271 ± 35 ms, p < 0.05; 10 µM REM: 280 ± 34 ms, p < 0.05) and significant shortening of the APD_90_ (baseline: 178 ± 31 ms; 1 µM REM: 142 ± 25 ms, p < 0.05; 5 µM REM: 137 ± 28 ms, p < 0.05; 10 µM REM: 131 ± 21 ms, p < 0.05). The dispersion of repolarization was not significantly altered (baseline: 54 ± 17 ms; 1 µM REM: 56 ± 18 ms, *p* = ns; 5 µM REM: 56 ± 22 ms, *p* = ns; 10 µM REM: 60 ± 21 ms, *p* = ns).

**Fig. 5:**
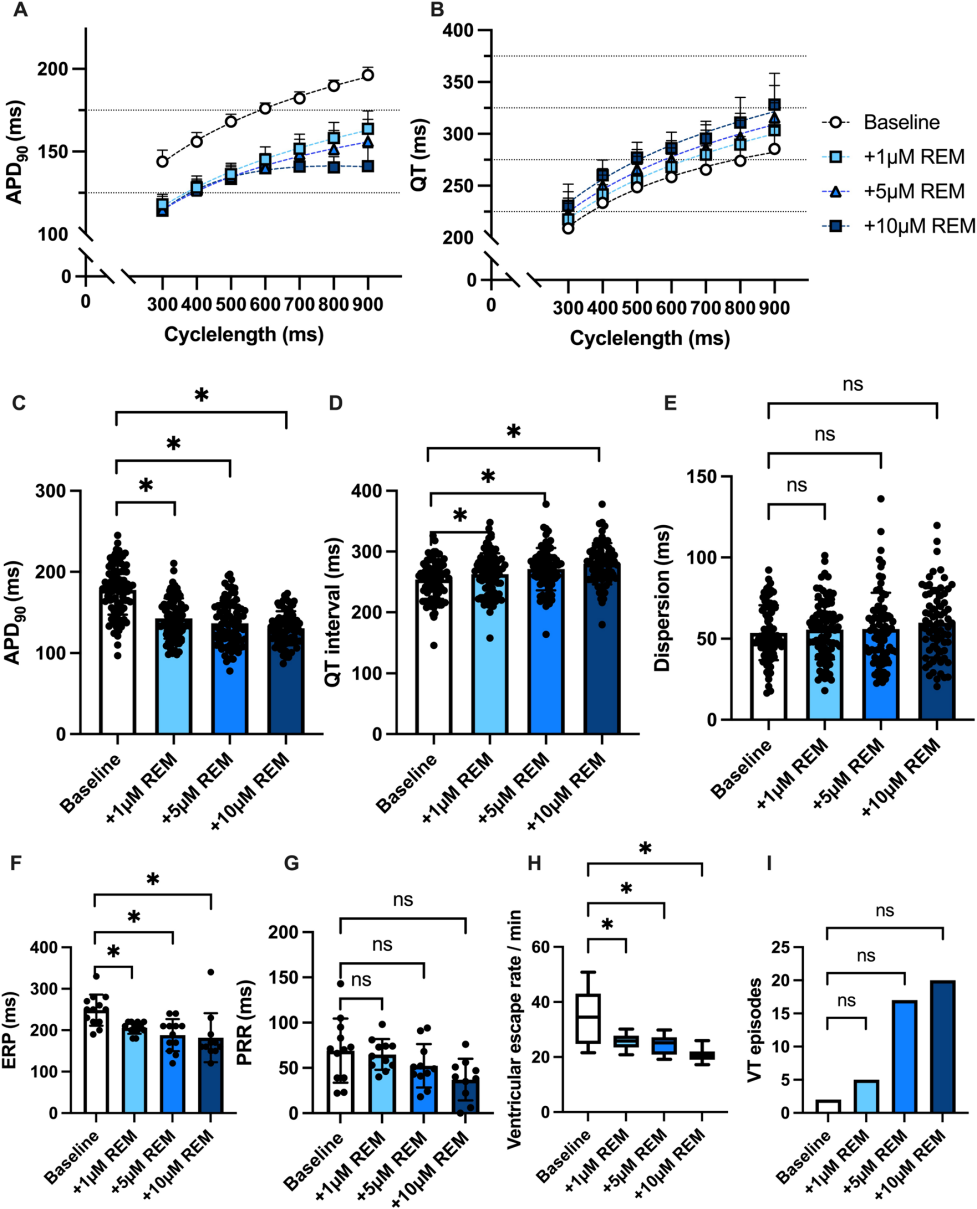
**A** Cycle length-dependent action potential durations (APD_90_) and **B** QT intervals under baseline conditions (empty circles) and after treatment with 1 µM (light blue square), 5 µM (blue triangle) and 10µM (dark blue square) remdesivir (REM). **C** Overall APD_90_ and **D** QT interval. Concentration-dependent effect of remdesivir on **E** spatial dispersion of repolarization, **F** effective refractory period (ERP), **G** post-repolarization refractoriness (PRR) and **H** ventricular escape rate. **I** Number of ventricular tachycardia (VT)/ fibrillation (VF) induced by programmed ventricular stimulation (* = p<0.05).

The effective refractory periods was significantly shortened under perfusion with remdesivir. The arrhythmia incidence was not significantly increased following addition of remdesivir to the perfusate (baseline: 2 episodes; 1 µM REM: 5 episodes, p = ns; 5 µM REM: 17 episodes, p = ns; 10 µM REM: 20 episodes, p = ns). The perfusion with remdesivir induced a significant bradycardia in the AV-blocked hearts. The effects of remdesivir on cardiac electrophysiology are summarized in Fig. 5.

## Discussion

### Electrophysiological Safety of Hydroxychloroquine

Hydroxychloroquine showed a significant QT prolongation when high doses (3 µM) were administered to the isolated rabbit hearts. This observation was paralleled by a significant increase in arrhythmia susceptibility following 3 µM hydroxychloroquine. In contrast to the QT interval, APD_90_ was not significantly prolonged compared to baseline. This effect is based on an increased dispersion of repolarization resulting in an increased T_peak_–T_end_ interval leading to an increased QT interval in the setting of an unaltered APD_90_.

The cellular electrophysiological effects of hydroxychloroquine reported previously are heterogenous. Different groups reported inhibitory effects of hydroxychloroquine and chloroquine on hERG-channels [7, 23, 24] resulting in APD prolongation and severe proarrhythmia [25] while the overall risk of arrhythmic events in patients treated with hydroxychloroquine seems to be low [8, 9]. The acceptable overall proarrhythmic risk of hydroxychloroquine might be clarified by the study by Borsini et al. [23] showing increased hERG protein transport in rabbit ventricular myocytes.

### Pharmacokinetic Suitability

The concentrations of hydroxychloroquine chosen in this study were in accordance with reported plasma concentrations and the recommended EC_50_ of hydroxychloroquine to inhibit SARS-CoV-2 in vitro [26]. Furthermore, as hydroxychloroquine is metabolized by CYP3A4, comedications with CYP3A4 inhibitors can lead to significantly elevated plasma levels [27].

## Electrophysiological Safety of Combining Hydroxychloroquine and Azithromycin

The addition of azithromycin caused a severe QT interval and APD prolongation. Furthermore, azithromycin induced severe dispersion of repolarization accompanied by a significantly increased arrhythmia susceptibility.

At the first glance these results might seem unexpected, as azithromycin did not show severe proarrhythmic effects and even antiarrhythmic effects when administered in combination with erythromycin in our model [13]. Nevertheless, QT prolonging and proarrhythmic effects of azithromycin are known [28] and especially the combination of azithromycin with hydroxychloroquine is known to inherit a severe proarrhythmic risk [10, 11].

Previous studies of our and other groups underlined the relevance of spatial dispersion of repolarization in drug-induced proarrhythmia by which the coexistence of excited and excitable cardiomyocytes give rise to ventricular arrhythmias [29], especially torsade de pointes tachycardia. The cellular mechanisms by which azithromycin causes these effects seems to be a combination of I_Kr_-inhibition and I_NaL_ induction [30].

Interestingly, sole azithromycin, in equivalent concentrations did not cause severe proarrhythmic effects in a previous study by our group [13] which suggests that the combination of the two potentially proarrhythmic drugs surpasses the repolarization reserve [31] resulting in proarrhythmia.

### Pharmacokinetic Suitability

The concentration of azithromycin was in accordance with our previous study of azithromycin on the Langendorff isolated rabbit heart. The plasma concentration of azithromycin following oral administration are significantly lower (0.4–1.1 µM) [30], but it was shown that azithromycin accumulates in cardiomyocytes resulting in local concentrations in the range of 100-200 µM [32].

## Electrophysiological Safety of Lopinavir

The perfusion with lopinavir induced slight, but non-significant changes of the APD_90_ while small but significant prolongation of the QT interval was observed. In accordance with the observations in the hydroxychloroquine group the prolonged QT interval results from a prolonged T_peak_-T_end_ ratio in the setting of an increased dispersion of repolarization. In a previous study lopinavir showed significant hERG-block with an IC_50_ of 8.5 µM [14]. Based on these results, we expected a more intense effect of lopinavir. However, physiological differences between single-channel studies and whole-heart models might be causative. Furthermore, I_Kr_ inhibition may well explain the increased dispersion. Because of the spatial heterogeneity of I_Kr_ endowment in cardiomyocytes, moderate I_Kr_ blockade in cells with low repolarization reserve may result in significant local APD prolongation with pronounced dispersion with stable overall APD.

### Pharmacokinetic Suitability

Concentrations of lopinavir were chosen based on antiviral activity in the studies of Choy et al. [33] and resulting plasma levels in the data of Alvarez et al. [34]. No comedication was performed with ritonavir, even though it is often used in clinical practice as a potent CYP3A4 inhibitor to enhance the effect of lopinavir. The omission of experimental combination with ritonavir was based on data from Soliman [35] and Sarapa [36] et al., who demonstrated no significant increase in QTc and no significantly increased arrhythmic risk with the addition of ritonavir. Nevertheless, the potent CYP3A4 inhibiting effect of ritonavir must be taken into consideration when combining lopinavir/ritonavir with other substances as for example hydroxychloroquine.

## Electrophysiological Safety of Remdesivir

Langendorff hearts perfused with remdesivir showed a slight QT prolongation, paralleled by a significant shortening of the APD_90_. Remdesivir led to a tendential increase in VT inducibility.

The opposite effect of remdesivir on QT time and APD was similarly reported by Pilote et al. [37]. They observed significant QT time prolongation in guinea pigs under remdesivir while APD was not significantly altered in Langendorff perfused hearts. The different observations regarding APD could be due to differences between species as well as the different cycle lengths of the measurements (200-350 ms in the Pilote Study [37] vs. 300-900 ms in our study). Remarkably, no AV-node ablations were performed in their Langendorff hearts.

The cellular effects of remdesivir are not conclusively understood and remain the subject of research. For example, Choi et al. [15] did not detect hERG inhibition in pluripotent stem cells while the group led by Al-Moubarak et al. [16] reported significant hERG blockade. Our results do not indicate significant hERG blockade under the clinically relevant (see below) concentrations used.

Most prominent was the significant bradycardia tendency with remdesivir, which has been widely reported from clinical practice [17–19] and from the Langendorff data of Pilote et al. [37]. The underlying mechanisms here are also not fully deciphered. Discussed are mitochondrial dysfunction [38] as well as activation of adenosine receptors via active metabolites [39] or remdesivir itself [37]. The acute bradycardic effects we observed after AV node ablation argue for acute interactions at the level of ventricular myocytes or the specific conduction system and against the involvement of transcriptional effects or active metabolites.

### Pharmacokinetic Suitability

The concentrations of remdesivir employed in this study are in accordance with the reported maximum plasma levels in humans following remdesivir administration of 2.5–9.0 µM [40, 41].

### Divergent Effects on APD_90_ and QT Intervals

In this study, different or opposing effects on APD_90_ and QT interval were observed in several groups, which may seem counterintuitive at first glance. However, this was observed more frequently by our [42] and other groups [43], as changes in dispersion or conduction velocity can cause QT time prolongation even with unchanged APD. Overall, other groups have already shown that isolated APD and QT interval are an insufficient parameters to assess drug-induced proarrhythmia [44].

## Limitations

Although our model does not allow the observation of active metabolites or transcriptional effects and undoubtedly limitations exist in translating the results from rabbit hearts to clinical application. Furthermore, as a result of the housing capacities only female reabbits were used, which are more prone to ventricular arrhythmias than male individuals [45].

Nevertheless, it is a very well established and accepted model for the evaluation of drug-induced proarrhythmia.

## Clinical Implications

In this model, different antiviral therapies showed divergent electrophysiological safety profiles. While proarrhythmic effects were especially observed following hydroxychloroquine and further aggravated when combining hydroxychloroquine and azithromycin, remdesivir and lopinavir showed rather safe electrophysiological profiles, although increased VT incidence was documented under all therapies. Nevertheless, in accordance with clinical observations, remdesivir showed significant bradycardic effects. The following clinical recommendations can be derived from these observations. All four agents tested here should be used with appropriate caution. Hydroxychloroquine, lopinavir, and remdesivir should not be used or should be used with extreme caution in patients with known QT prolongation or QT prolonging comedications. Hydroxychloroquine should not be combined with azithromycin because of a significant proarrhythmic effect. When using remdesivir, the bradycardic potential should be considered. Overall, further clinical studies are needed to assess the risk of relevant bradycardia in patients. In the case of marked conduction delays or syncope, special caution seems to be warranted.

## Data Availability

The data are available from the corresponding author on reasonable request.
